# Spindle biochemistry responds to compressive force from the nuclear envelope to tune spindle dynamics during closed mitosis

**DOI:** 10.64898/2026.01.15.699759

**Published:** 2026-01-15

**Authors:** Taylor Mahoney, Christopher Needham, Reem Hakeem, Marcus Begley, Christian Pagán Medina, Mary Williard Elting

## Abstract

During closed mitosis in *S. pombe* , the nuclear envelope and mitotic spindle must work together mechanically and biochemically to ensure successful nuclear division. Previous work has demonstrated that mechanical force from the nuclear envelope, transmitted through spindle pole bodies, can re-shape the spindle. However, it remains unclear how force might alter spindle biochemistry. Here, we investigate how force reprograms the spindle with two approaches: chronically increasing nuclear envelope tension via the lipid synthesis inhibitor cerulenin, and acutely applying force through an optical trap. Both perturbations slow elongation dynamics and reduce microtubule number. Despite this reduction, key spindle proteins Ase1 and Klp5 increase their density at the spindle midzone, indicating inward force from the nuclear envelope can alter spindle biochemistry. We find that while motor proteins Klp5 and Klp6 only minimally affect the spindle’s response to increased nuclear envelope force, the combination of removing Ase1 and increasing nuclear envelope force together rescue spindle stability. Together, our findings reveal that nuclear force on the spindle does not merely alter its shape, but is key in regulating its biochemistry to maintain force balance.

## Full Text Availability

The license terms selected by the author(s) for this preprint version do not permit archiving in PMC. The full text is available from the preprint server.

